# Pandemic consequences: An increase in divorce rate, domestic violence, and extramarital affairs at the time of COVID-19 pandemic: A sad Persian story

**DOI:** 10.3389/fpubh.2022.1100149

**Published:** 2022-12-13

**Authors:** Amir Khodavirdipour, Mahdi Samadi

**Affiliations:** ^1^Department of Biology, Faculty of Natural Sciences, University of Tabriz, Tabriz, Iran; ^2^Pubic Health and Women's Rights Activist, Tabriz, Iran; ^3^Farshchian Sina Hospital, Hamadan University of Medical Sciences, Hamedan, Iran

**Keywords:** post pandemic, COVID-19, psychosexual, domestic violence, honor killing

## Introduction

### Domestic violence and extramarital affairs at the time of the COVID-19 pandemic

COVID-19 was caused by the SARS-CoV-2 virus, which originated from a Wuhan fish market and then quickly spread around the world (1, 2). One of the major impacts of COVID-19, besides health and financial situations, is that family cohesion is shaken, which can be determined by the increased rate of divorce rate, extramarital affairs, and domestic violence due to prolonged duration of lockdown against preventing COVID-19 transmission (3). Researchers in a latest study demonstrated that housewives faced several problems during the quarantine phase such as lifestyle imbalance, life with fear and anxiety, personal health problems, Internet addiction, and low mental health (4–6). Based on Iran's registar office for birth, death, marriage, and divorce datasheet from 2012 to date, the statistics show a rising trend in divorce incidents and a decline in the marriage rate despite the 2 years of corrections (7). Domestic violence against women in the Middle East is nothing new; needless to say, the situation in Iran is far better than a few neighboring countries and the Persian Gulf countries and North Africa, where 30–64% (country-wise) of male participants hinted to have caused domestic violence and carried out sexual harassment (8). In the year 2020, Iraq and Iran have been ranked as the world's angriest countries (9) while the latest local survey brings to the spotlight that 46% of women in Iranian families experienced domestic violence, which can be divided into sexual and physical abuse, intimidation, isolation, and economic abuse, by men using male privileges including honor killing, reportedly with more than 8,000 registered cases from 2010 to 2014 (10).

### Psychosexual health impacts of COVID-19 in Iran: An inside view

The divorce trend in Iran has been rapidly increasing for the last 10 years, which makes Iran one of the countries with the highest and unprecedented increase in divorce (11), especially since the COVID-19 pandemic (7). The top underlying reasons for divorce in Iran are marital infidelity, extramarital affairs, sexual dissatisfaction, addiction-associated domestic violence, and growth in women's competence (high level of education, seeking independence, and fighting for their basic rights including battling against forced complementary hijab), and all together are reasons for Muslim men to instigate financial and emotional abuse, which consequently results in extreme control (12). Tehran, Fars, Esfahan, and East-Azerbaijan, respectively, had the highest rate of divorce during the pandemic based on the reports from the civil registration office (7). The other reason for the divorce rate to be high is the enforcement of male-friendly laws in this culture in this regard. The husband can initiate a divorce without the wife's consent. Furthermore, it is the wife who will face extreme measures of intolerable social behavior after the divorce besides losing child custody as Sharia Law often gives custody to the father. All this endless power can further embolden a husband who without any social opprobrium indulges in extramarital affairs or uses domestic violence for any rightful or unjust things in his belief and the wife has no right to any sort of objection. The social construction of male dominance as a sign of triumph is created by religion, social norms, and value systems that can then provide key justification initially for extramarital affairs and domestic violence and then for more extreme behaviors such as divorce or honor killing. The epicenter of this lifestyle-cum-culture and the hub of the above said acts are the Middle East and North Africa. The rate of divorce and domestic violence and extramarital affairs are higher in countries with tighter Islamic laws and harsher penalties such as death for extramarital affairs, despite religious leaders' denials that this violence has a strong root in the past (13). For instance, the Iranian Panel code article 630 permits a man to kill his wife and her sex partner if he witnesses the act of their sexual intercourse. Another example, article 301 specifies that the parental grandfather and the father will not face retaliation for killing the child (14). Then, 4 months into the COVID-19 pandemic, three shocking domestic violence cases which ended in murder (honor killing) drastically shook the world in 20 days: Romina Ashrafi, Fatemeh Barhi, and Rayhaneh Ameri on 21 May, 14 June, and 15 June 2020, respectively, in the north, south-west, and south-east of Iran (15). All the three murderers escaped heavy sentences by applying for article 301. By expanding the issue, we can notice that divorce, extramarital affairs, domestic violence, and sometimes honor killing and murder are multifactorial, including low social status, rapid modernization, poverty, and being religious extremists as key factors of such incidents (14). In addition, we should not forget about Afghan refugee women in Iran who confronted many problems along with dealing with COVID-19 due to their fragile conditions (16).

## Concluding remarks

Desperation, anger, unemployment, and poverty shall liable a man to brutality. Lockdown policies and the COVID-19 situation could lead to considerable vulnerability of people facing barriers and people with incomes below the poverty line, which can have a more destructive consequence than the pandemic itself. Increased stress, financial insecurity, poor economic conditions, isolation, and restricted access to personal space at home besides weak socioeconomic status lead an individual to unstable mental health. Modernization speed in Iran and education regarding civil rights need to be at the same speed; we encourage the Iranian government, which was always supportive in similar situations, to set up special hotlines and expert advocacy teams in each state to help women from various backgrounds across the country to legally protect the women rights. In addition, we advise future research on this important factor, which may affect other aspects of life directly or indirectly.

## Author contributions

All authors listed have made a substantial, direct, and intellectual contribution to the work, and approved it for publication.
